# The Flight Pattern of Navel Orangeworm (*Amyelois transitella* Walker) 2008–2023 in California Pistachio

**DOI:** 10.3390/insects15120919

**Published:** 2024-11-25

**Authors:** Joel Philip Siegel

**Affiliations:** Commodity Protection and Quality Unit, San Joaquin Valley Agricultural Sciences Center, United States Department of Agriculture, Agriculture Research Service, Parlier, CA 93648, USA; joel.siegel@usda.gov; Tel.: +1-559-596-2735

**Keywords:** navel orangeworm, *Amyelois transitella*, pistachio, flight dynamics, biofix, population dynamics

## Abstract

The navel orangeworm, *Amyelois transitella*, is the principal pest of pistachio and almond in California. The timing of the insecticide application is challenging because there is no model that predicts when pistachio is vulnerable to infestation. Sixteen years of pistachio flight data from Madera and Fresno counties (541,892 adults) were analyzed to determine if there was a consistent starting point each year for flights that overlap pistachio vulnerability. This effort was complicated by changes in trap lures over this period, as unmated females were replaced by a combination lure consisting of the synthetic pheromone and phenyl propionate, which is needed because mating disruption suppresses pheromone lure trap capture. There were two additional complications: the increased degree day accumulation during the growing season and the three-fold hectarage expansion of pistachio. A biofix at 944.4 degree days °C from the beginning of the year was identified from the dataset, which was consistent across all years in both counties. Using the biofix, subsequent flight peaks occurred at 277.78 degree day °C intervals (generation time on new crop pistachios), corresponding to three weeks in the field. This biofix can be used to improve the timing of field scouting, which in turn will improve the timing of insecticide application.

## Simple Summary

The navel orangeworm, *Amyelois transitella*, is the principal pest of pistachio and almond in California. The timing of insecticide applications is challenging because there is no model to predict when pistachio is vulnerable to infestation; therefore, it is difficult to decide when to intensify scouting orchards when making application decisions. Sixteen years of flight data were analyzed (541,892 adults) to determine if there was a predictable pattern in flight activity from the beginning of the year. A pattern was found using degree days, a measurement of heat accumulation, with the count starting at the beginning of the year. The goal is to be able to scout orchards to identify pistachio vulnerability before peak insect pressure. A common starting point was identified that occurred 944.4 degree days °C from 1 January each year. This starting point, also known as a biofix, remained consistent despite the changes in trap lures and the expansion of almond and pistachio hectarage that occurred over the 16-year period. This information can be used to predict flights each year, thereby increasing scouting efficiency because nut vulnerability can be determined before peak pressure.

## 1. Introduction

The navel orangeworm, *Amyelois transitella* Walker (Lepidoptera: Pyralidae), is the primary pest of California almond (*Prunus dulcis;* 81% of the world supply) and pistachio (*Pistacia vera*; 54% of the world supply) and is an important pest of walnut (*Juglans regia*) [1,2]. Currently, these crops are planted on >900,000 ha in California and have a combined annual farm gate value exceeding US $9 billion, and in 2020, pistachios contributed $5.2 billion to the California economy [3]. This multivoltine moth has a mutualistic relationship with fungal species belonging to the genus *Aspergillus* that produce aflatoxins; consequently, feeding causes direct damage to the nuts and can cause indirect damage by mycotoxin contamination [4,5,6,7].

The population dynamics of this moth are complex because it develops on both the new crop and mummies (unharvested nuts from the previous crop) and has different generation times on mummies and new crop nuts, as well as on almond and pistachio, developing fastest on new crop pistachio [8,9,10,11]. *A. transitella* readily moves between orchards [12,13,14,15] and the proximity of both crops in the San Joaquin Valley, combined with different crop maturity, increases its regional abundance [16]. The greatest overlap of *A. transitella* cohorts occurs from August through September, when the generation time is the shortest due to a combination of rapid degree day accumulation and the availability of high nutritional quality new crop nuts.

Egg traps were initially used to monitor *A. transitella* [17,18], but their use decreased when a pheromone lure became available in 2013 [19,20,21]. Pheromone lures are now widely used, but their attractancy is suppressed in orchards using mating disruption, which can also affect trap capture in orchards as far away as 3 km from the dispensers [22]. Phenyl propionate (PPO) is an alternative lure that works in orchards using mating disruption [23]. This lure is commercially available and successfully captures both males and mated females and is usually used in tandem with a pheromone lure [24,25,26].

**Figure 1:**
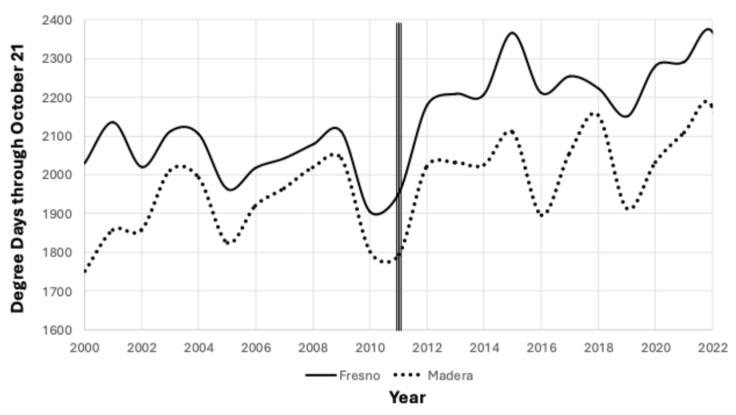
Comparison of degree day °C accumulation 1 January–21 October 2000–2022, for Fresno and Madera counties, California.

*A. transitella* was traditionally managed by a combination of sanitation, which is the timely removal and subsequent destruction of mummies, and insecticide sprays. Nonpareil almonds, the first new crop variety of almonds to become vulnerable to infestation, are typically sprayed early to mid-July. Kerman variety pistachios are typically sprayed late August through early September (first shake) and late September through early October (when there is a second shake). Pistachios become vulnerable when they ripen and their hull breaks down, exposing the split shell [27,28,29]. Mating disruption became part of the pest management plan for many orchards after 2015 [16]. The tolerance to *A. transitella* damage in tree nuts varies; the almond goal is damage < 2% and the pistachio goal is more stringent, damage < 0.99% [30]. In some years, there are *A. transitella* outbreaks with a >five-fold damage increase; 2017 was a high damage year for all tree nuts, with damage exceeding 20% in some loads of almonds and pistachios [30]. Damage has increased since 2012 due to the combination of increased host availability (currently 640,000+ ha almonds and 245,000+ ha pistachios) and beginning in 2012, increased heat unit accumulation during the growing season [31], as seen in Figure 1.

A major challenge for successful insecticide application in pistachio is spray timing because hull breakdown in pistachio is not predictable. This study identifies consistent peaks in *A. transitella* flight activity that occur when new crop pistachios are vulnerable to infestation so that scouting can be intensified during these periods to determine whether insecticide applications are necessary, and if so, ensure that they are applied in time. The flight peaks were initially identified in Madera County, California, using trapping data collected from 2008–2023, and then validated using trapping data from Fresno County 2021–2023. These data include adults captured using three types of lures during the study period (unmated female, pheromone, pheromone + PPO).

## 2. Materials and Methods

### 2.1. Study Locations

Trapping was conducted at two locations in Madera County between 2008–2023. The first location was Agri-World Cooperative (31545 Donald Avenue, Madera, CA 93636, USA), a 1295-ha pistachio orchard containing mature Kerman variety pistachios, where trapping was conducted from 2008–2023. The second location was S&J Ranch (39639 Avenue 10, Madera, CA 93636, USA), where trapping was conducted on 400 ha from 2008–2013. Trap capture was pooled for both locations from 2008–2013 (286,360 males); from 2014 onwards, only Agri-World Cooperative was used (122,756 adults), for a total of 409,116 adults captured during the study. In Fresno County, two 246 ha pistachio orchards located near Five Points (an unincorporated community) were used from 2021–2023. These orchards served as controls for a sterile navel orangeworm release program that is a collaborative effort between the United States Department of Agriculture, the California Department of Food and Agriculture (CDFA), and the University of California [26]. A total of 132,776 moths were trapped and these data are reported by CDFA on their webpage (https://www.cdfa.ca.gov/plant/ipc/nowp/index.html, accessed on 1 September 2024).

### 2.2. Lure and Trap Types

From 2008–2012, unmated females originating from a laboratory colony in Parlier, CA were used as lures, with 3 females placed in a single mesh bag per trap, replaced twice weekly [32]. *A. transitella* was reared in incubators or temperature-controlled chambers at 30 °C, 16:8 h light/dark photoperiod, using a modified wheat bran diet [10]. Only males were captured in the traps and the data were pooled to provide weekly totals for trap capture. From 2013–2017, the traps used a synthetic pheromone lure to catch males instead (Suterra, Bend, OR), and from 2020–2023, a combination of two synthetic lures consisting of a PPO and a pheromone lure (Pherocon NOW L2, Trece, Adair, OK, USA) were used in both Madera and Fresno counties, trapping males and mated females [24,25]. Suterra wing traps (Suterra, Bend, OR, USA) were used in Madera County, and Pherocon 1C wing traps (Trece, Adair, OK, USA) were used in Fresno County; traps were checked at weekly intervals. The trap density in Madera County was one trap per 80 ha, while the trap density in Fresno County was one trap per 7.2 ha [26]. In Madera County, trapping began on 1 April and ended on 21 October, while in Fresno County, trapping began on 22 February 2021, 14 February 2022, and 20 March 2023. The delay in 2023 was caused by flooding in the fields. A cutoff date of 21 October was used for Fresno County, although trapping continued through December.

### 2.3. Degree Day Calculation

For Madera County, degree days were calculated using the UC IPM degree day calculator (https://ipm.ucanr.edu/weather/weather-models/#gsc.tab=0, accessed on 1 September 2024) utilizing data from CMIS station #145, 2008–2011. These calculations utilize the daily high and low temperatures and generate a sine curve using a lower threshold of 12.778 °C and an upper threshold of 34.444 °C. After 2011, the degree days for Madera County were calculated using a Spectrum Watchdog Series 2000 ministation (Spectrum Technologies, Aurora, IL, USA) placed at a height of 1.8 m in the canopy. The Spectrum ministation calculates degree quarter-hours instead of degree days using the actual temperature instead of the daily high and low temperatures. The degree days for Fresno County were calculated using the UC degree day calculator utilizing data from Five Points A (CMIS #2). The degree days used in Figure 1 were calculated using the UC degree day calculator and the temperature data for Firebaugh/Aliso for Madera County and Parlier CMIS #39 for Fresno County.

### 2.4. Graphing the Start and End Points and Determining the Flight Intervals

Degree day calculation started at the beginning of the year, 1 January, and ended on 21 October, which is the date processors usually stop receiving pistachio shipments. The activity period of interest begins in mid to late July, when the first new crop pistachios, known as “early splits”, become available for infestation by *A. transitella* [33]. Initially, four years of flight data from 2008–2011 were collected as a starting point to determine the peaks based on visual inspection of the weekly trap capture. The start for the first peak was set at 944.4 degree days °C (1700 degree days °F) from 1 January, with 277.78 °C increments thereafter (500 degree day °F intervals), corresponding to the average pistachio generation time for new crop nuts based on laboratory and field studies [10]. The complete values of interest from July onwards are 944, 1222, 1500, 1778, and 2056 degree days °C from 1 January (1700, 2000, 2700, 3200 degree days °F), although 2056 degree-days °C was not important during the early years of this study. During the period 2008–2011, there was a single pistachio harvest that ended in late September at approximately 1600 degree days °C, but as the crop increased in value, the pistachio industry added a second harvest (second shake) that typically began in late September and usually continued into mid-October. Consequently, the timing of the late September and October peaks in flight activity became more important to determine when additional sprays were needed to protect the late harvest [29].

### 2.5. Validation

The data for 2008–2011 were inspected as described above and the means for the predicted peaks of 944, 1222, and 1500 were calculated ± SD. These values were considered acceptable if the actual peak occurred within two days (an interval of 39 degree days °C based on peak accumulation of 19.4 degree days per day from late July onwards) of the predicted peak. Similar calculations were made for the years 2012–2015 and 2020–2023 in Fresno and Madera counties. The years 2016–2019 were omitted because the early peaks were suppressed by mating disruption.

### 2.6. Type of Lures Used and Data Grouping

Madera flight data was analyzed in groups for the periods 2008–2012 (unmated females as lures), 2013–2015 (pheromone lure), and 2021–2023 (pheromone + PPO lure). The data for 2013 were included, although trapping ended September 30 due to the Federal government shutdown. The Fresno data for 2021–2023 were used to validate the projected flight peaks for two reasons. First, these traps employed both types of synthetic lures and were more likely to trap adults when mating disruption was used, and second, these traps were deployed at a higher density than the Madera County traps, which also increased their likelihood of catching moths.

## 3. Results

During the 16 years studied, there was a shift in weekly trap capture over time (Table 1), before mating disruption (2008–2015) and after mating disruption (2016–2023). After 2015, adult capture in July, calculated as the percentage of total capture from July through 21 October, decreased by 57.6% to 10.1%, while in September, it increased by 34.2% to 45.1%. Percent capture was relatively unchanged for August and October. The most likely explanation for this change is that mating disruption suppressed capture in July, although some adults were still caught, and its effect lessened in September due to a combination of population increase and immigration into pistachio from neighboring almond orchards being harvested.

**Figure 2:**
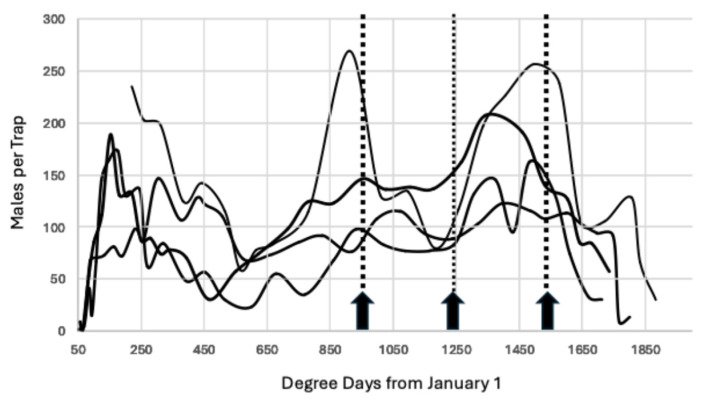
Average males per trap 2008–2011 through 21 October using virgin females as the lure, Madera County. The arrows mark increments of 277.78 degree days beginning at 944.4 degree days °C from 1 January.

When the trap data for 2008–2011 were superimposed, a consistent peak occurred at 944.4 degree days °C, although its magnitude varied by year, as seen in Figure 2. Using this as a starting point or biofix, when additional peaks are predicted using 277.78 degree day °C intervals, there were three peaks for the period of July through 21 October.

A comparison between the predicted flight peaks and actual peaks observed is reported in Table 2.

The greatest deviation occurred for the predicted late peak at 1778 degree days °C for both Fresno and Madera counties, with the actual peaks occurring three to four days earlier. For the other time points, the observed peaks were within two days of the predicted peaks, with the closest fit at 1222 degree days °C.

The increase in degree day accumulation beginning in 2012 did not alter either the starting point of 944.4 degree days °C or the subsequent projected peaks for 2012–2015, Figure 3. Note that the number of predicted peaks increased from three to four for all years, and there was a predicted fifth peak in October for two years.

The trap data for 2021–2023 used both types of synthetic lure (2021–2023) and caught males and mated females, but the pattern of flight peaks did not change, as seen in Figure 4, although trap capture in 2023 ended sooner than the other years. However, trap capture was higher in 2023 because that was an outbreak year in almonds and the standing population increased in all counties that grew almonds.

The degree day accumulation for 2023 was similar to total degree days for 2008–2011 because the “atmospheric river” that occurred in California broke the 10-year pattern of increased degree day accumulation from 2012. This return to a lower degree day accumulation is illustrated in Figure 5, which compares the trap capture for 2010 and 2023. The peaks are similar through 1650 degree days °C from 1 January, but there was one additional peak in 2023 compared to 2010, which cannot be explained.

Degree day accumulation varied over the 16-year period studied. The year 2014 had the greatest accumulation of degree days, 2308, and the year 2010 had the lowest accumulation of degree days, 1695, a difference of 613, which is sufficient for an additional two generations to have developed in 2014, Figure 6. However, when trap capture is compared, the flight peaks are congruent through the first 1500 degree days °C from 1 January.

The data for Fresno County confirm the pattern observed in Madera County. Figure 7 reports the total adult weekly capture per 258 ha block instead of the average capture per trap. The biofix remained at 944.4 degree days °C with subsequent peaks at 1222.2, 1500, 1777.8, and 2055.6. The dotted line is the trap capture for the outbreak year of 2023, and capture was six-fold higher at 1500 degree days °C than the previous two years. Trap capture also peaked at 1500 degree days in Madera County, Figure 5.

## 4. Discussion

The goal of this study was to determine if there are predictable patterns in flight activity that can be used to improve spray timing, which is a different goal than monitoring flight activity to determine if an insecticide spray can be skipped [34]. Spray timing is an ongoing issue because the *A. transitella* damage has increased since the start of this study in 2008. There were outbreaks in 2012, 2013, 2016, 2017, 2023, and 2024 (pistachios only in 2013 and 2016, and almonds only in 2023 and 2024) due to a combination of increased host availability and degree day accumulation during the growing season. During the period 2008–2023, pistachio hectarage rose from 79,434.9 to 245,013.7 ha, a 3.08-fold increase, while almond hectarage rose from 321,725.1 ha to 631,309.6 ha, a 1.96-fold increase [3,35,36]. Commodity prices have fluctuated during this period, and the factors mentioned above combined with the recent reduction in the price of tree nuts make it more challenging to control *A. transitella*. Almond and pistachio also pose unique challenges since the period of nut vulnerability at hull split varies. There is a temperature-based predictive model for almond hull split (https://fruitsandnuts.ucanr.edu/Weather_Services/almond_hullsplit_prediction/, accessed on 1 September 2024), but the breakdown of pistachio hull integrity cannot be predicted; therefore, there is more uncertainty surrounding the optimal timing of pistachio sprays. Generally, pistachio hull breakdown begins in early September, but in some years, hull breakdown occurs as early as late July (2017). In contrast, the vulnerability of Nonpareil almonds begins in late June. The uncertainty surrounding the optimal timing of insecticide sprays due to nut physiology is exacerbated by the common challenge of getting the owner/orchard manager to begin spraying when advised by their pest control advisor.

It is important to recognize that management decisions play an important role in determining the extent of both pistachio and almond damage during the season, and simple tools are needed to guide these decisions. This last point was the foundation for choosing 944.4 degree days °C as the starting point or the biofix. California growers use degree days °F in their calculations, and a biofix set at 1700 degree days °F (944.4 °C) is a round number and easy to remember. This flight peak typically corresponds to the period of maximum *A. transitella* pressure for Nonpareil almonds during July, although the date ranged from 29 June through 1 August for the two extremes in this study. This predicted biofix does not have to be an exact match for the flight peak because pest control advisors and orchard managers typically monitor their traps weekly, therefore a prediction that is within two days of the actual peak is useful. Since insecticide application should be made before the flight peak so that the nuts are coated before oviposition occurs, the bias should be directed toward an earlier biofix. The subsequent intervals of 277.78 °C also do not have to be a perfect fit for the subsequent peaks, since this information is used to scout an orchard and get orchard sprayers ready. These projected degree day intervals may also be adjusted backward for orchards by pest control advisors when there is a consistent delay in their client implementing their recommendations, to ensure that scouting or insecticide applications are made on time, although I do not recommend deviating beyond 100 degree days °C (7 days) on the early side. It is far better to be two or three days early for an application than two or three days late.

In Table 2, the greatest disparity between the predicted peak of 944 occurred in Madera County from 2021–2023, but this difference (14.8 degree days) of 1.6% is less than one day. The greatest difference for the next interval of 1222 (12.2 degree days) was 1%, and the greatest difference for the interval of 1500 (35) was 2.3%, corresponding to the peak occurring less than two days earlier than predicted. The greatest discrepancy of 2.9% occurred for the final peak of 1778 (51.7 degree days), with the actual peak occurring three days earlier than predicted, as illustrated in Figure 7. This last peak may be harder to predict because of the extensive emigration from almonds and subsequent immigration into pistachio during the September and early October harvest of pollenizer almond varieties.

**Figure 3:**
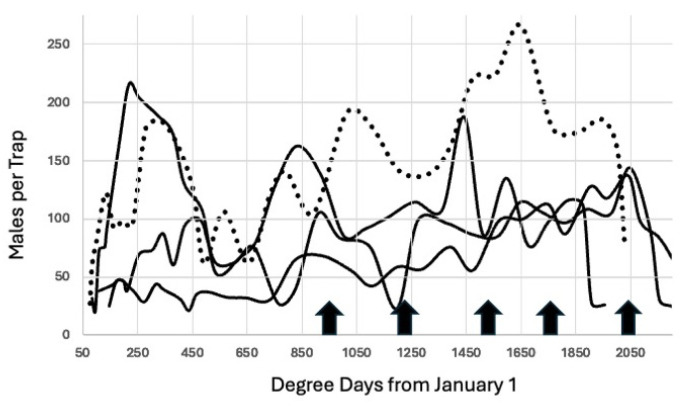
Average males per trap 2012–2015 through 21 October using virgin females (2012, 2013) and synthetic pheromones (2014, 2015) as lures, Madera County. The arrows mark increments of 277.78 degree days beginning at 944.4 degree days °C from 1 January. The dotted line represents the adults captured in 2013, an outbreak year in pistachio, and the solid lines are for the other years.

**Figure 4:**
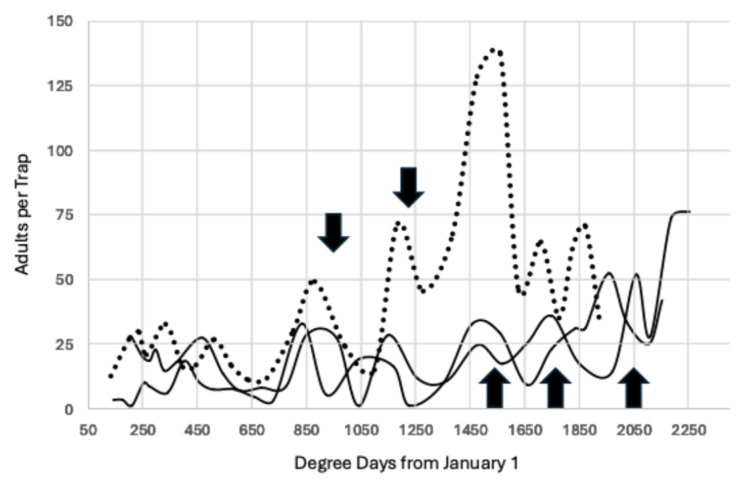
Average moths per trap 2021–2023 through 21 October using a combination of synthetic pheromone and phenyl propionate lures, Madera County. The arrows mark increments of 277.78 degree days beginning at 944.4 degree days °C from 1 January. The dotted line represents the adults captured in 2023, an outbreak year in almonds, and the solid lines are for 2021 and 2022.

**Figure 5:**
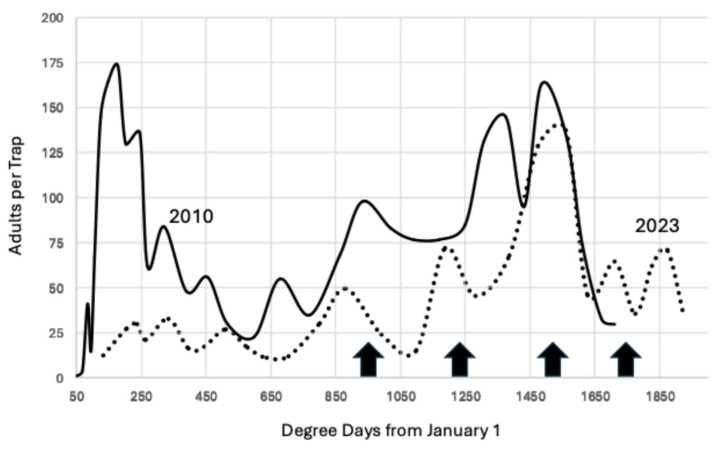
Average moths per trap through 21 October for 2010 and 2023, Madera County. In 2010, traps used unmated females and in 2023, traps used a combination of synthetic pheromone and phenyl propionate lures. The arrows mark increments of 277.78 degree days beginning at 944.4 degree days °C from 1 January. The dotted line represents the adults captured in 2023, an outbreak year in almonds, and the solid line is for 2010.

**Figure 6:**
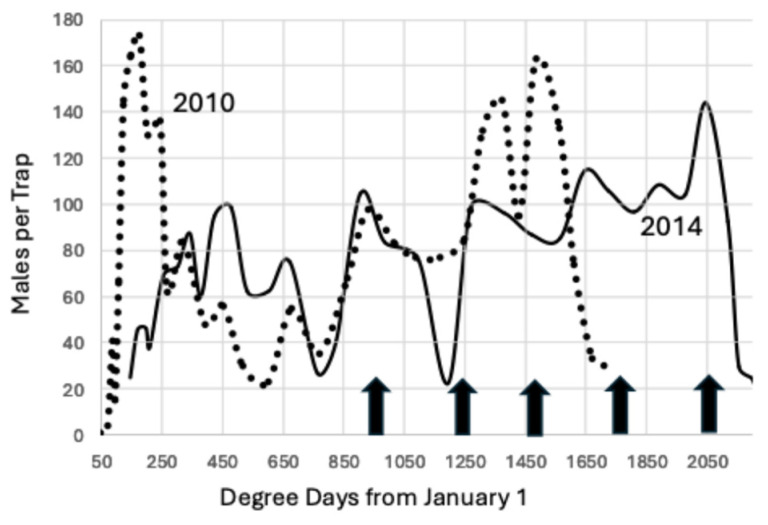
Average males per trap 2010 (dotted line) vs. 2014 through 21 October using unmated females as a lure in 2010 and a synthetic pheromone lure in 2014, Madera County. The arrows mark increments of 277.78 degree days beginning at 944.4 degree days °C from 1 January. The dotted line represents the adults captured in 2010, the year with the lowest degree day accumulation in this study, and the solid line is for 2014.

**Figure 7:**
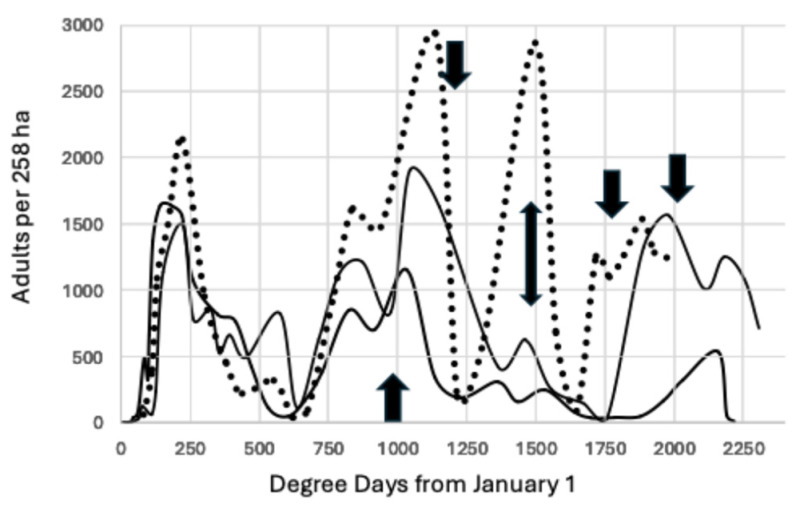
Total moths per 258.9 ha block, 2021–2023 through 21 October, Fresno County, using a combination of synthetic pheromone and phenyl propionate lures. The arrows mark increments of 277.78 degree days °C beginning at 944.4 degree days from 1 January. The dotted line represents the adults captured in 2023, an outbreak year in almonds, and the solid lines are for 2021 and 2022.

During the course of this study, the number of generations of *A. transitella* per season increased over time. Initially, there were three generations of *A. transitella* during the period 2008–2011, as seen in Figure 2. The rise in degree day accumulation that began in 2012 (Figure 1) added an additional generation to the season, and in some years, there were two additional generations, as illustrated in Figure 3, Figure 4, Figure 5 and Figure 6. However, the biofix of 944.4 degree days °C from 1 January remained constant, even when flight data for both sexes were included for the period 2020–2023 (Figure 4 and Figure 7). Mated females are attracted to the phenyl propionate lures and the combination of the two lures attracts both sexes of *A. transitella* to the traps. Male and female capture are highly correlated [26]; therefore, the use of phenyl propionate does not introduce bias. There were five predicted peaks for Fresno and Madera counties for 2020–2023, and the actual flight peaks were a reasonable match to predicted peaks, with the exception of the last peak, Table 2.

An Ideal indicator is unaffected by changes in trap lure necessitated by the adoption of mating disruption and is also unaffected by changes in degree day accumulation during the season. There are two figures that compare the data for 2010, the year with the lowest degree day accumulation, to other years. The first comparison, Figure 5, demonstrated that the flight dynamics in 2023 reverted to the pattern of 2010, yet the biofix was the same (944) despite the difference in the lures used (unmated female vs. synthetic pheromone + PPO). The second comparison, Figure 6, contrasted 2010 with 2014, the year that had the highest degree day accumulation. There was excellent agreement for the biofix and the next two intervals of 1222.2 and 1500 degree days °C, although the lures differed (unmated females in 2010 and synthetic pheromone in 2014). There also was a difference of 567 degree days °C between these two years, sufficient for two additional generations to develop in 2014. The close match in flight activity for the earlier peaks between these two extreme years demonstrates that the biofix of 944.4 degree days °C was robust.

In conclusion, the goal of this study was to determine if there were consistent patterns in *A. transitella* flight activity that could be used to improve its control in pistachio. The analysis of 16 years of flight data from Madera County (409,116 adults) and three years of flight data from Fresno County (132,776 adults) found a consistent pattern of flight peaks when 944.4° degree days °C was used as the biofix, and that flight peaks occurred at intervals of 277.8 degree days °C thereafter. The challenge is determining the best way to use this information to refine current *A. transitella* management. There are a variety of strategies listed for spray timing including egg traps combined with degree days based on the generation time for development in mummies (583.3 degree days °C), and insecticide application based on the proposed start of harvest. This study demonstrated that the new crop pistachio generation time (277.78 degree days °C) starting with degree day accumulation from 1 January can be successfully used to predict flight peaks. Given the cost of labor and the large pistachio hectarage planted, it is more cost effective to schedule orchard scouting using degree days calculated from 1 January than using a combination of egg traps and the mummy generation time. Focusing on the mummy generation time is misleading because the development of *A. transitella* on new crop nuts is 52.4% faster [9,10]. Ultimately, it is nut vulnerability that determines pistachio damage, and the best use of the proposed peak flight timing is to improve the scheduling of orchard scouting and to motivate the orchard manager to ensure that the insecticide and orchard sprayers are available when an insecticide application is necessary

## Figures and Tables

**Table 1:** Average percent capture (±SD) for the months of July–21 October before mating disruption (2008–2015) compared to capture using pheromone + phenyl propionate lures (2021–2023).

Years	July	August	September	October
2008–2015				
	23.8 ± 7.2	26.7 ± 9.1	33.6 ± 6.3	16.0 ± 10.7
2021–2023				
	10.1 ± 8.5	28.0 ± 8.5	45.1 ± 5.9	16.3 ± 6.1

**Table 2:** Mean ± SD degree days °C (DD) from 1 January for the predicted navel orangeworm flight peaks 2008–2023 from Fresno and Madera counties.

Years	944 DD	1222 DD	1500 DD	1778 DD
2008–2011	946.8 ± 38.0	1218.0 ± 48.4	1500.3 ± 21.4	
2012–2015	940.6 ± 48.0	1234.2 ± 39.1	1465.0 ± 14.8	1785.2 ± 35.4
2021–2023	958.8 ± 33.8	1203.9 ± 48.7	1470.6 ± 9.3	1735.1 ± 21.7 ^a^
2021–2023 ^b^	946.0 ± 27.8	1232.0 ± 82.0	1496.7 ± 33.0	1726.3 ± 58.0 ^a^

^a^ Peaks occurred more than two days earlier than predicted. ^b^ Fresno County data.

## Data Availability

All data are available in this paper upon request and at the website listed.
